# Evaluation of re-used medicinal leeches as a potential source for nosocomial MDR bacterial infections in canines

**DOI:** 10.3389/fvets.2025.1649736

**Published:** 2025-10-31

**Authors:** Mariajesus Soula, Kara Berke, David A. Upchurch, Laura A. Barbur, Abigail L. Huber, Raghavendra G. Amachawadi

**Affiliations:** ^1^Department of Clinical Sciences, Kansas State University, Manhattan, NY, United States; ^2^Friendship Hospital for Animals, Washington, DC, United States

**Keywords:** leech application, canine, MRSA—methicillin-resistant *Staphylococcus aureus*, wound, infection

## Abstract

**Objectives:**

Leech therapy is commonly used in medicine as a treatment for venous congestion. Since a concern with reusing leeches is potential spread of infections, it is recommended to discard leeches after use. If a leech harbored bacteria from one patient in its gastrointestinal (GI) tract, it could transmit them to another patient, potentially, serving as a vector for multidrug resistant (MDR) infections. The objectives of this study were to determine if MDR *Staphylococcus aureus* can be transmitted from inoculated blood into a leech, how long can the bacteria can persist within the leech and its environment, and if leeches can transmit the bacteria during refeeding.

**Animals:**

63 leeches were split into eight treatment groups and one control group.

**Methods:**

Treatment leeches were fed canine blood inoculated with an MDR strain of *Staphylococcus aureus* while control leeches were fed clean canine blood. Cultures were obtained at 1 day, 1 week, and 1-, 2-, 3- and 4-months post-inoculation. Culture samples were taken from the aquarium water, GI contents, and blood that the leeches were allowed to refeed on. Cultures were evaluated for the presence of *Staphylococcus aureus.*

**Results:**

All water samples were negative except for one tank at 7 days after feeding. After 2 and 3 months, all GI tracts and blood meal samples were negative, respectively.

**Clinical significance:**

Leeches will harbor MDR *Staphylococcus aureus* after inoculation. This bacterium is not detectable in the water after 7 days or in the leech and blood meal after 3 months. Further studies should be conducted to determine the reproducibility of these results given the novel complications identified throughout the course of our study.

## Introduction

Medicinal leeches (*Hirudo medicinalis* and *verbena*) have been used for thousands of years for treatment of skin diseases, nervous system abnormalities, urinary and reproductive system problems, inflammation, and dental problems (1). In modern human medicine, they are predominantly used to help improve the venous flow of congested skin or muscle flaps or wounds following a surgical reconstruction or repair. Scientists have discovered over 100 different active compounds within the leech saliva, including hirudin. These compounds work together and with the host tissues to create an anti-inflammatory and anti-coagulant environment, as well as exerting anesthetic and analgesic effects.

Leeches have also crawled their way into veterinary medicine for the same purposes, most commonly for oncological patients following reconstructive skin flaps post mass removal. Other described uses for veterinary patients include osteoarthritis, neuropathies, eczema, polycythemia vera, and mastitis among many others (2–5). Despite evidence of efficacy, leech therapy is still not common in veterinary medicine. One reason for their scarcity in veterinary medicine might include the recommendation to sacrifice medicinal leeches after a single use. In human medicine, re-using leeches is forbidden due to the concern of cross contamination of bacteria from infected patients. If leeches can harbor a potentially infectious bacteria in their gastrointestinal (GI) tract and then transmit that to a patient, it is possible that if a leech were to feed on a patient with a multidrug resistant bacterial (MDR) infection, it could harbor that MDR bacterial species and, when placed on a different patient, could potentially transmit that infection. Additionally, it is unknown if they can transmit bacteria through the water column of their housing system.

Single use of leeches in veterinary medicine may not be practical due to the cost associated with purchasing new leeches for each patient and the time for leeches to be shipped from their source to the veterinary hospital. For example, without factoring in hospital markup, the cost of overnight shipping 5 leeches for a patient was approximately $277.75 at the time this manuscript was prepared (Leeches USA Ltd., Westbury, NY, United States). Since leeches only feed once every 3 to 6 months, if a veterinary hospital could set up a leech colony and reuse each leech this may increase the attractiveness of this therapeutic modality.

To the authors’ knowledge, there are currently no studies evaluating the harboring or transmission of MDR bacteria in leeches. A bacterial species of particular interest is MDR *Staphylococcus aureus*. This bacterium is one of the leading nosocomial pathogens in humans with global public health significance because it shows a very virulent and a multi-drug resistant pattern (6–9). It is known that 94% of these strains are resistant to penicillin via the production of penicillinase, an enzyme that breaks down the antimicrobial agent (10). Other strains have been found to be resistant to different antimicrobials based on specific antimicrobial resistance genes. For example, the methicillin resistant *S. aureus* strains (MRSA) express the *mecA* gene which encodes for penicillin-binding protein. A previous study in Germany found that MRSA was isolated in 17.8% of veterinary patients sampled compared to the human outpatient prevalence of 5.4% (11). There is an increasing prevalence in the isolation of MRSA from both human and veterinary medicine settings as well as continued increase in resistance (12). Because this bacterium is frequently isolated in veterinary medicine patients and has zoonotic potential, we chose to use this organism as our test species.

The primary objective of this study was to determine if MDR *Staphylococcus aureus* can be transmitted from inoculated blood into a leech and, if so, how long can it persist within the leech and its aquatic environment. A secondary objective was to determine if inoculated leeches could transmit the bacteria during refeeding. We hypothesized that leeches inoculated with an MDR strain of *Staphylococcus aureus* would not harbor or transmit the MDR bacterium 6 months after inoculation. Additionally, we hypothesized that MDR *Staphylococcus aureus* would not be isolated from the water of their environment or inoculated into fresh blood during feeding.

## Materials and methods

### Study population

Based on preliminary data and expected variability, a power analysis determined that eight replicates per time point would be sufficient to detect statistically significant differences with a power of 0.8 and *α* = 0.05. To account for potential sample attrition due to leech mortality, we maintained a separate aquarium containing reserve leeches under identical environmental conditions, ensuring that any losses could be replaced without compromising the required number of replicates at each time point. Accordingly, nine 5-gallon aquaria were established to house seven leeches each, with one leech designated per time point and one extra as a backup. Eight aquaria comprised the inoculated study population, while one served as the sterile control. All aquaria were maintained at conditions exceeding current husbandry standards, which typically recommend storing leeches in containers of water in a refrigerator (Leeches USA Ltd., Westbury, NY, United States). Instead, our leeches were kept in filtered aquaria, providing a higher standard of care. Sterile leeches were obtained from a medical supplier (Leeches USA Ltd., Westbury, NY, United States), shipped overnight, and allowed a two-week acclimation period in their randomly assigned treatment aquaria prior to experimental manipulation.

### *Staphylococcus aureus* preparation

#### Selection and preparation of *Staphylococcus aureus* culture for inoculation

A *Staphylococcus aureus* clinical isolate (2022–5#3SA) from a canine was selected owing to its niche as a commensal and known to reside in skin, soft tissues, and/or superficial infections. The isolate, 2022–5#3SA, is multidrug resistant with resistance determinants to more than three classes of antimicrobials. The isolate 2022–5#3SA was confirmed to be multidrug resistant based on phenotypic susceptibility testing and genomic analysis. Specifically, it exhibited resistance to tetracycline, daptomycin, quinupristin and dalfopristin and gentamicin antimicrobials. Our study aimed to evaluate the transmission dynamics of MDR *S. aureus* within the leech model using a clinically relevant isolate. Since 2022–5#3SA itself was the MDR strain under investigation, it served as the experimental strain rather than a control. A single isolated pure colony of *Staphylococcus aureus* from a blood agar plate was inoculated into 10 mL of Mueller-Hinton broth and incubated overnight at 37 °C. Colonies selected for further use were medium-sized, round, golden-yellow, and consistent with typical *S. aureus* morphology. One hundred microliters of the overnight broth culture were inoculated into a fresh 10 mL Mueller-Hinton broth and incubated for 3–4 h at 37° C to reach an absorbance of 0.4 at 600 nm [~1 × 10^8^ colony forming unit (CFU)/ml]. This step was chosen to maintain consistency with antimicrobial susceptibility protocols. Serial dilution and plate counting were done to enumerate the bacterial concentration present in the inoculum and also to achieve the inoculum dosage for our experiment. We were trying to achieve approximately 1 × 10^8^ colony forming unit (CFU)/ml for meal preparation and to infect leeches. For most of the sensitivity assays, the standardized inoculum has a concentration of 1–2 × 10^8^ and this is the basis of selecting this bacterial concentration. The inoculum concentration of 10^8^ CFU/mL was selected based on prior studies demonstrating that this level reliably establishes colonization in invertebrate models without causing immediate lethality or compromising host viability. Our primary objective was to assess the persistence and transmission potential of MDR *S. aureus* within the leech gastrointestinal tract, and a standardized inoculum allowed for consistent comparison across time points and experimental conditions.

### Meal preparation and leech inoculation

Fresh whole blood from a canine was obtained from the hospital’s blood donor program at the start of the project and again at the 3-month time point. The blood was from a clinically healthy dog without any abnormalities on physical exam or on complete blood work that would indicate an underlying infectious agent within the blood.

Discussion with leech keepers recommended using blood sausages as feeding vessels for the leeches. A commercially available sausage casing was purchased. Ten grams of blood were weighed out and nine individual blood sausages were prepared by filling a segment of casing with the blood and tying knots at both ends to make a tightly packed sausage. Eight of these were then inoculated with 1 × 10^8^ CFU/mL culture of *S. aureus* and used for feeding leeches from the treatment groups. A ninth sausage was prepared in the same manner but not inoculated and this was fed to the control group.

After the initial feeding, the remaining prepared sausages not fed to the leeches, were stored in a freezer kept at a temperature of 0° F. The exact number of sausages needed were then removed from the freezer and placed in the refrigerator (kept at 35° F) a day prior to the next round of trials in order to allow them to thaw.

### Feeding

Leeches were assigned randomly to either the control group or one of the eight study groups. All members of each group were placed in a dry container containing a blood sausage that was either contaminated with *S. aureus* (treatments) or uncontaminated (control). Leeches that did not attach to the blood sausage and were not observed to visibly eat were offered the blood from the inside of the sausage poured onto a sterile sample cup lid and placed in the dry container. All leeches were observed to feed either from the blood sausage or from the container for at least 20 min. The leeches were allowed to feed until satiated. Satiation was defined as the point in time where a leech that could be observed to be actively feeding (active contractions) for at least 20 min detached itself from the feeding source and was subjectively noted to be at least 2xs its original size.

### Sampling

Samples obtained from each aquarium included water from the system, an individual leech’s GI contents, and newly fed blood to simulate feeding on a new patient. All samples were submitted for aerobic culture and susceptibility. The laboratory where the experiment was conducted was certified by the International Biosafety Committee and was cleared as a BSL-2. As such, all sampling and sterilization protocols were reviewed by the committee and strictly adhered to by the authors to prevent contamination.

At each sampling point, samples were first obtained in a sterile fashion from the control system. Once these samples were obtained and put away, the study aquaria were all sampled. Gloves and instruments were changed between each sample. Surfaces were wiped with disinfectant between each sample.

### Water sampling

A sterile culture tube was dipped into the aquarium to obtain a water sample from each system at 1 day, 1 week, 1 month, and 2 months post-feeding. Sampling of aquarium water was discontinued for each aquarium after negative culture results were obtained in that aquarium at two subsequent time points. A sample from each system was also taken as a control the day prior to feeding after the leeches had been living in their aquaria for a 2 week period.

### Leech GI tract sampling

At 1 day, 1 week, 1 month, 2 months and 3 months after feeding, a leech was randomly selected from each aquarium. The leech’s GI tract was sampled by gently massaging the animal from caudal to cranial to make it regurgitate and then collect the contents of its crop as it was produced from its mouth using sterile swabs. Once a leech was sampled, it was placed in a separate holding unit (a smaller container with water and small holes for flow) within their current system to keep them separate from the unsampled leeches. This was done to prevent sampling the same leech twice while keeping the group together to see if transmission to the environment occurred. The original methodology called for sampling of leech GI tracts to be discontinued after negative culture results were obtained at two subsequent time points (similar to the aquarium water). However, three positive leeches at the one-month time point were noted to be from aquaria that tested negative at the one-week time point. Additionally, at the two-month time point, a single leech tested positive from an aquarium that tested negative at the one-month time point. Due to concerns that not all leeches within an aquarium were equivalently infected with *S. aureus*, every leech in each aquarium was sampled at both 3 and 4 months. Leeches were each separated and fed individually as previously described to avoid cross contamination between leeches.

### Fresh blood meal sample

To determine if leeches could transmit bacteria to a new patient, the same leech whose GI tract was sampled at each time point was allowed to feed from ~2 mL of non-inoculated blood that was placed in a sterile sample cup lid. The leech was allowed to feed until satiated. Once satiated, the remnant blood in the feeding container was swabbed and collected for culture using sterile swabs. This was done at 1 day, 1 week, 1 month, 2 months and 3 months after feeding. Similar to the leech GI tract sampling, the original methodology called for sampling of fresh blood meals to be discontinued after negative culture results were obtained at two subsequent time points. It was noted at the one-week time point that positive fresh blood meals were obtained from two of the aquaria that tested negative at the one-day time point. Additionally, at the one-month time point, it was noted that a positive fresh blood meal was obtained from an aquarium that was negative at the one-week time point. For this reason, for the 3^rd^ and 4^th^ month time points, every remaining leech in each system was fed a fresh blood meal and these were all sampled.

### Isolation and identification of *Staphylococcus aureus*

The leech gut contents, water, and the patient sample were enriched in Mueller-Hinton broth (Becton and Dickson, Sparks, MD) with 6.5% sodium chloride (Sigma-Aldrich, St. Louis, MO) at 37 °C for 24 h. The enriched suspension was then inoculated onto Mueller-Hinton and blood agar plates and incubated at 37 °C for 24 h (23). Putative *Staphylococcus aureus* colonies were selected from the agar plates and subjected to catalase and coagulase tests. The species confirmation were done by PCR detection of *staph* (756 bp) and *nuc* (279 bp) genes. The confirmed *Staphylococcus aureus* isolates were stored in Protect beads (Cryo-Vac®, Round Rock, TX) at −80 °C for further use.

### Antibiotic susceptibility determinations

Minimum inhibitory concentrations (MICs) were determined by broth-microdilution method as per CLSI guidelines (2023). The MIC for *S. aureus* isolates were determined by the broth micro-dilution method using the Sensititre® automated antimicrobial system (Trek Diagnostics Systems, Cleveland, OH). National Antimicrobial Resistance Monitoring System Gram-positive panel plates (CMV3AGPF) were used with the aid of the Sensititre® automated inoculation delivery system (Trek Diagnostics Systems, Cleveland, OH). Appropriate ATCC (American Type Culture Collection, Manassas, VA) quality control strains; *Enterococcus faecalis* ATCC 29212, and *Staphylococcus aureus* ATCC 29213 were used as reference standards for susceptibility testing. The MIC for each isolate were recorded and classified as resistant, intermediate, or sensitive based on the Clinical Laboratory Standards Institute (CLSI, 2023) guidelines.

### Statistical analysis

All statistical analyses were performed using Stata version 16.0 (StataCorp LLC, College Station, TX, United States). Descriptive statistics were used to summarize the distribution of *Staphylococcus aureus* across timepoints and sample types. Pairwise comparisons were conducted using chi-square tests of independence to evaluate differences in *S. aureus* prevalence between categorical groups. To evaluate the effect of timepoint and sample type on the binary outcome (presence/absence of *S. aureus*), multivariable logistic regression models were fitted with categorical predictors. Odds ratios (OR) with 95% confidence intervals (CI) were reported. Predicted probabilities of *S. aureus* detection were estimated using the margins command and visualized with marginsplot to illustrate the relationship between timepoints and sample types. Model fit was assessed using the Hosmer–Lemeshow goodness-of-fit test, with non-significant results (*p* > 0.05) indicating adequate fit. Predictive performance was evaluated using the receiver operating characteristic (ROC) curve and the area under the curve (AUC) statistic, where values closer to 1.0 indicated stronger discriminatory ability. All statistical tests were two-tailed, and a *p*-value < 0.05 was considered statistically significant.

## Results

### Overall prevalence of *Staphylococcus aureus*

A total of 195 samples were analyzed for the presence of *Staphylococcus aureus*. Among these, 27 samples (13.9%) tested positive, while 168 samples (86.2%) were negative. The prevalence of *Staphylococcus aureus* varied significantly across timepoints (likelihood-ratio χ^2^(5) = 34.99, *p* < 0.001). At timepoint 0, 30.6% (11/36) of samples were positive, and prevalence remained similar at timepoint 1 with 29.6% (8/27) positive. Prevalence declined to 18.5% (5/27) at timepoint 2 and further decreased to 11.1% (3/27) at timepoint 3. No positive samples were detected at timepoints 4 (0/42) or 5 (0/36). Overall, these findings indicate a significant downward trend in *S. aureus* detection over time, with complete absence after timepoint 3. The temporal and group-wise distribution of *S. aureus* across sample types, including blood, gastrointestinal swabs, and water, collected from control and treatment groups is summarized in Table 1.

**Table 1:** Prevalence of *Staphylococcus aureus* in various sample types collected from different groups across several timepoints.

Sample type	Timepoint	Control		Treatment	
		No. of samples tested	No. of positive samples (%)	No. of samples tested	No. of positive samples (%)
Pre water	0	1	0 (0)	8	0 (0)
Blood	0	1	1 (100)	8	6 (75)
	1	1	0 (0)	8	3 (37.5)
	2	1	0 (0)	8	4 (50)
	3	1	0 (0)	8	3 (37.5)
	4	2	0 (0)	19	0 (0)
	5	2	0 (0)	16	0 (0)
GI swab	0	1	0 (0)	8	4 (50)
	1	1	1 (100)	8	3 (37.5)
	2	1	0 (0)	8	1 (12.5)
	3	1	0 (0)	8	0 (0)
	4	2	0 (0)	19	0 (0)
	5	2	0 (0)	16	0 (0)
Post water	0	1	0 (0)	8	0 (0)
	1	1	0 (0)	8	1 (12.5)
	2	1	0 (0)	8	0 (0)
	3	1	0 (0)	8	0 (0)

### Prevalence across sample types

The prevalence of *Staphylococcus aureus* differed across sample types, although the overall association did not reach statistical significance (likelihood-ratio χ^2^(8) = 13.80, *p* = 0.087). Blood samples showed the highest prevalence, with 23.9% (16/67) positive, followed by gastrointestinal swabs with 11.9% (8/67) positive. Control blood and control GI swab samples each had 12.5% positivity (1/8). Postwater samples showed a lower prevalence of 4.2% (1/24), while no positives were detected in prewater samples (0/8), additional postwater samples (0/8), postwater controls (0/4), or prewater controls (0/1). Although blood samples tended to have a higher proportion of positives compared to other sample types, the difference did not achieve statistical significance at the 0.05 level.

Logistic regression analysis was performed to assess the association between *Staphylococcus aureus* presence and sample type/timepoint (*n* = 96). The overall model was significant (LR χ^2^(7) = 25.37, *p* = 0.0007) with a pseudo R^2^ of 0.22, indicating that the predictors explained about 22% of the variation in *S. aureus* occurrence. Compared to blood samples, the odds of detecting *S. aureus* were significantly lower in GI swabs (OR = 0.29, 95% CI: 0.09–0.90, *p* = 0.032) and postwater samples (OR = 0.03, 95% CI: 0.003–0.26, *p* = 0.002). Blood control and GI swab control samples showed lower odds as well, though these associations were not statistically significant (*p* > 0.05). Across timepoints, the odds of *S. aureus* detection decreased, with timepoint 3 showing a significant reduction (OR = 0.12, 95% CI: 0.02–0.59, *p* = 0.009), while timepoint 2 showed a trend toward significance (OR = 0.25, 95% CI: 0.06–1.06, *p* = 0.060). Timepoints 4 and 5 had no estimable odds due to absence of positive cases. The constant term (OR = 2.84, *p* = 0.078) indicated a marginal baseline likelihood of *S. aureus* detection. In summary, *S. aureus* was significantly less likely to be detected in GI swab and postwater samples compared to blood, with a notable decline in detection by timepoint 3.

### Water samples

**Figure 1:**
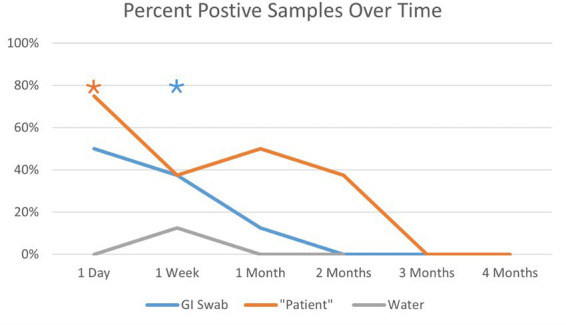
A simple line graph demonstrating the percentage of positive results by sample type over the time points. The orange asterisk and the blue asterisk represent a time point with a positive control sample.

There were a total of 36 samples, which included one sample from each system prior to inoculation obtained as a baseline. All samples from study aquaria tested negative for *Staphylococcus aureus* at baseline. All systems tested negative at all time points tested except for one of the treatment aquariums at 7 days post-leech feeding. Sampling was discontinued after the two-month samples were obtained due to persistently negative results across all groups (Figure 1; Table 2).

**Table 2:** Percentage of positive samples by time point and sample type.

Sample	1 Day	1 Week	1 Month	2 Months	3 Months	4 Months
GI Swab	50%	*37.5%	12.5%	0%	0%	0%
Blood meal	*75%	37.5%	50%	37.5%	0%	0%
Water	0%	13%	0%	0%	-	-

*indicate a positive control sample at that time point.

### GI content and “blood meal” samples

There were a total of 67 samples. Nine samples, one from each system, were obtained at 1 day, 1 week, and one-month post-inoculation. GI samples from one aquarium tested positive at day one, negative at 1 week, and then positive again at 1 month. This also occurred for the blood meal samples from a different aquarium. For the remainder of the blood meal samples, there were no positive samples until the 2-month time point. For these reasons, at the 3 month and four-month sample dates, all leeches from each system were sampled for a total of 67 leeches from the treatment group and two from the control group. This led to some leeches being sampled a second time. Due to an unfortunately high rate of leech mortality due to escaping from confinement between sampling periods and the trauma of stripping, we had fewer treatment and control individuals at the three- and four-month time points. The results from these samples are summarized in Figure 1 and Table 3. There were a total of two positive control samples (one GI sample and one blood meal sample). For both groups, there were no positive samples starting at 2 and 3 months, respectively (Figure 1; Table 2).

**Table 3:** Total sample quantities for both the GI and blood meal sample groups and the total positive samples for the GI and blood meal sample groups by time point.

Time point	Samples	GI positives	Blood meal positives
1 Day	8	4	6
1 Week	8	3	3
1 Month	8	1	4
2 Months	8	0	3
3 Months	19	0	0
4 Months	16	0	0

Sample quantities increased at time points 3 and 4 months since the protocol was changed to sample every individual animal, three animals died between time points 3 and 4 months.

## Discussion

Leech therapy provides a plethora of benefits to a patient ranging from local anesthetic to improving local perfusion. Among the numerous compounds found in their saliva, hirudin, antistastin, and ghilanten produce a synergistic inhibitory effect on clotting factor Xa (13). These effects remain for up to several hours after the leech has finished feeding. While the blood meal consumed by a leech actively aids in the improvement of venous stasis, the persistence of the effect of these compounds allows for continued bleeding from the leech bite, further decreasing congestion and improving microvascular flow (14–17). A recent study found that medicinal leech therapy resolved 75% of venous congestion lesions in small animal patients (18).

As discussed, the concern for reusing leeches is transmission of bacterial infections. Because leeches have a symbiote bacteria, *Aeromonas hydrophila,* which has been reported to be transmitted to ~18% of human patients undergoing leech therapy (19), human patients are prophylactically treated with antibiotics to prevent such infection (20). However, wounds for which leeches are used in veterinary medicine may have an MDR bacterial infection. This could be detrimental to another patient if a leech were to become contaminated and then spread that infection.

Because data has shown that certain species of leeches have antimicrobial properties in their saliva, it may be possible that leeches will not harbor MDR *Staph. aureus*. For example, a study conducted by Abdualkader et al. (2011) showed that leech saliva extract has antibacterial activity against *Staphylococcus aureus, Salmonella typhi,* and *Escherichia coli* (21). This research proposes that the antibacterial properties of saliva extracted from Malaysian leeches (a non-medicinal leech species) are novel but could vary from species to species. Furthermore, it has been shown that Gram-negative and Gram-positive inhibitory activity may vary from species to species of leech. Other research shows that the peptides theromacin and theromyzin, from the coelomic liquid of leeches, have antibacterial activity against Gram-positive bacteria rather than Gram-negative bacteria (22). This suggests that future studies should further evaluate the antibacterial activity in the saliva and gastrointestinal extracts from leeches of various origins, including those typically used for medicinal purposes.

In this study, 12% of the GI content samples from the leeches obtained by stripping of the crop were positive for MDR *Staphylococcus aureus.* No GI samples tested positive after one-month post inoculation. However, 23% of the blood meal samples tested positive and it was not until the three-month time point that there were no more positive blood meal samples. These results indicate that, while we could did not obtain a positive culture from the leech GI contents, the leech was still harboring the bacteria and transmitting it to a new and clean blood meal. We speculate that this occurred because the leeches were fed the new blood meal first and then were stripped. In doing this, they may have deposited their GI bacteria into the meal (causing them to decrease their load) and then when they consumed more sterile food, they further diluted their bacterial load. Future studies could look to enumerate the load in the GI tract of the leeches to see if this speculation is correct.

Taken together, these findings demonstrate that *S. aureus* was most prevalent in blood samples, particularly at early timepoints, and significantly less likely to be detected in GI swabs and post-wash water samples. The prevalence declined sharply over time, with no detection observed beyond timepoint 3. The inclusion of both control and treatment groups across multiple timepoints strengthens the reliability of observed trends and supports the temporal association between intervention and reduction in *S. aureus* prevalence. The complete absence of *S. aureus* detection beyond timepoint 3 suggests either effective microbial clearance or suppression, potentially linked to treatment or environmental factors. The consistent absence of *S. aureus* in pre- and post-wash water samples across all timepoints underscores the effectiveness of sanitation protocols and suggests minimal risk of waterborne transmission in this setting.

It is important to note, however, that the study design assumed that all were inoculated with bacteri, which cannot be conclusively proven with this study design. Testing the GI samples involves stripping the leeches. Performing this is traumatic and also does not allow for the investigator to control how much of volume of GI content is sampled. We were unable to test if the leeches had all been inoculated because sampling immediately after feeding of the inoculant could have resulted in a loss of all of the in negative results at subsequent sampling times solely due to the immediate post-feeding sampling event. It is also important to note that a negative result immediately after feeding would not guarantee that no bacteria entered the leeches’ GI tracts because of the antimicrobial properties of the leeches’ mouths and saliva.

In order to determine if MDR *Staphylococcus aureus* could be shed and persist in the aquatic environment after the leeches were fed, we sampled the water from the aquaria at various time points. This is clinically important to determine since hungry and satiated leeches can be kept together and we wanted to know if there was potential risk to the people handling these animals or among conspecifics. We found that when inoculated with MDR *Staphylococcus aureus,* a positive culture was only obtained from one aquarium at one-week post-inoculation yielding an overall 2% positive rate. This makes it extremely unlikely that the bacteria are being transmitted to other leeches or humans via the water. If not all leeches were inoculated with bacteria as assumed, leech to leech transmission via the water may have been possible and would not be possible to detect with this study design. It is also possible that, despite strict sterile technique, this sample was positive due to a sampling error, either via incidental contamination during sampling or lab handling error.

Throughout the sampling period, there were a total of two positive control samples, one GI sample and one blood meal sample. These results are believed to be due to contamination of samples, given that the control leeches were sourced from a lab that supplies them to human hospitals and that they were never fed prior to being obtained and did not feed on inoculated blood. Since there were a few other positive samples and given that very strict biohazard protocol was followed throughout this experiment, these positive samples are believed to be erroneous at these time points.

Initially, the protocol was to discontinue sampling when two negative time points were achieved for each group. It was, however, noted that two treatment groups tested negative and then subsequently tested positive. This was attributed to the fact that a different leech from each group was sampled each time between the time points 1 day through 2 months. We hypothesize that perhaps not every leech became inoculated at the inoculation feeding and thus an individual could have sampled negative while others in the same aquarium were positive. Future studies should consider weighing each individual leech before and after feeding to ensure that all leeches consumed enough for inoculation. Because this difference in results across time points was noted, it was elected to begin to sample each individual leech from each system to obtain serial negative results for each animal (Table 3). Future studies could also consider maintaining all leeches in individual containers and testing each leech at each time point until there are two negative samples for that individual.

Unfortunately, several leeches escaped over the course of the study and so we had fewer treatment and control individuals at the three- and four-month time points. This resulted in fewer leeches at later sampling times than needed to achieve desired results based on the power analysis. Additionally, while sampled leeches should be housed in a way that allows them to be identified from the unsampled individuals, it is not recommended to maintain them in smaller containers within their system. We found that doing so meant that if a leech was to vomit post-feeding, the water within the container was not filtered as easily as the remainder of the system due to the smaller container having to be made secure with smaller holes to prevent the leeches from escaping. This led to the death of several of our sample leeches and decreased numbers at the three- and four-month time points.

*Staphylococcus aureus* was used for this study due to its zoonotic potential. While we have demonstrated that this particular species is no longer detectable in the blood meal after 3 months post-inoculation, there are many other harmful bacterial species that could be found on a small animal patient and potentially spread. Further studies with different bacterial strains need to be performed to further assess the safety of reusing medicinal leeches in small animal patients or if additional prophylactic antibiotic measures should be taken. Additionally, it should be evaluated if a patient with an infected wound that is receiving Hirudu therapy experiences anti-microbial benefits from the saliva of healthy leeches.

Leeches consume 5–15 mL of blood per meal. Another limitation of our study was that leeches were not allowed to feed to satiation since we needed them to be hungry again for sampling. It is possible that the reduced food intake of the leeches could have affected the bacterial load they consumed as compared to leeches feeding on clinical patients. However, in a clinical setting, if a leech is allowed to feed until satiation when being used for patient therapy, it will likely not want to eat for the next 6 months as noted by the author’s experience. This means that by the time the leech is ready to go to work again, the risk of MDR *Staphylococcus aureus* transmission is minimal.

In conclusion, leeches inoculated with MDR *S. aureus* do not harbor the bacteria or transmit it via feeding beyond 3 months after inoculation. While a single positive sample was obtained, it is unclear whether this was sample contamination. While we would like to assume that water from the aquaria housing inoculated leeches do not have positive cultures post-inoculation and thus is not a concern for zoonotic infection, this particular aspect needs to be further evaluated. Further studies need to be performed with other veterinary pathogens to ensure transmission of these pathogens do not occur. Veterinary hospitals can use this information to start a leech colony, with the size of the colony based on the demand of that hospital such that leeches can be reused only after every 3 months. This will allow for a more cost-effective way to provide this treatment option and enable greater opportunity to provide treatment to more patients. This colony can be kept with as simple of a setup of two aquaria with filtration. In one aquarium, hungry leeches are housed. When these leeches are used, they are moved to a separate system for satiated leeches. This will ensure that there are always leeches available at the hospital and that the hospital can set a more affordable fee to “rent out” the leeches per treatment. If there are a low number of cases requiring leeches, the hungry leeches can be fed portions of beef liver or other types of intermediate snacks. While the study provides valuable insights into temporal and sample-type-specific prevalence, future investigations incorporating molecular typing and larger control cohorts could further elucidate strain-specific dynamics and resistance profiles.

## Data Availability

The raw data supporting the conclusions of this article will be made available by the authors, without undue reservation.

## References

[1] Munshi Y Ara I Rafique H Ahmad Z. Leeching in the history – A review. Pak J Biol Sci. (2008) 11:1650–3. doi: 10.3923/pjbs.2008.1650.1653, PMID: 18819614

[2] Buote NJ. The use of medical leeches for venous congestion. Vet Comp Orthop Traumatol. (2014) 27:173–8. 24764080 10.3415/VCOT-13-10-0122

[3] Canpolat İ Sağlam N. Treatment of diffuse hematoma in a dog with the medicinal leech, *Hirudo medicinalis*. Fırat Üniversitesi Doğu Araştırmaları Dergisi. (2004) 2:97–9.

[4] Canpolat İ Sağlam N. Treatment of aural hematomas in dogs with medicinal leech, *Hirudo medicinalis*. Fırat Üniversitesi Doğu Araştırmaları Dergisi. (2004) 2:67–9.

[5] Net CS Arnoldq P Glaus TM. Leeching as initial treatment in a cat with polycythaemia Vera. J Small Anim Pract. (2001) 42:554–6. doi: 10.1111/j.1748-5827.2001.tb06027.x, PMID: 11721985

[6] Kitti T Boonyonying K Sitthisak S. Prevalence of methicillin-resistant *Staphylococcus aureus* among university students in Thailand. Southeast Asian J Trop Med Public Health. (2011) 42:1498–504. 22299421

[7] Pantosti A Venditti M. What is MRSA? Eur Respir J. (2009) 34:1190–6. doi: 10.1183/09031936.00007709, PMID: 19880619

[8] Peacock SJ Paterson GK. Mechanisms of methicillin resistance in *Staphylococcus aureus*. Annu Rev Biochem. (2015) 84:577–601. doi: 10.1146/annurev-biochem-060614-034516, PMID: 26034890

[9] Prenafeta A Sitjà M Holmes MA Paterson GK. Short communication: biofilm production characterization of mecA and mecC methicillin-resistant *Staphylococcus aureus* isolated from bovine milk in Great Britain. J Dairy Sci. (2014) 97:4838–41. doi: 10.3168/jds.2014-7986, PMID: 24881796

[10] Scherrer D Corti S Muehlherr JE Zweifel C Stephan R. Phenotypic and genotypic characteristics of *Staphylococcus aureus* isolates from raw bulk-tank milk samples of goats and sheep. Vet Microbiol. (2004) 101:101–7. doi: 10.1016/j.vetmic.2004.03.016, PMID: 15172692

[11] Feuer L Frenzer SK Merle R Leistner R Bäumer W Bethe A. Prevalence of MRSA in canine and feline clinical samples from one-third of veterinary practices in Germany from 2019–2021. J Antimicrob Chemother. (2024) 79:2273–80. doi: 10.1093/jac/dkae225, PMID: 39007221

[12] Shoaib M Tahir Z Shabbir MZ Aqib AI Farooq U Abbas T. MRSA compendium of epidemiology, transmission, pathophysiology, treatment, and prevention within one health framework. Front Microbiol. (2023) 13:1067284 36704547 10.3389/fmicb.2022.1067284 PMC9871788

[13] Dynowski Zygmunt Franciszek. Podstawy Hirudoterapii. (2007). Warszawa: Elsevier.

[14] Weinfeld AB Yuksel E Boutros S Gura DH Akyurek M Friedman JD. Clinical and scientific considerations in leech therapy for the management of acute venous congestion: an updated review. Ann Plast Surg. (2000) 45:207–12. doi: 10.1097/00000637-200045020-00021, PMID: 10949353

[15] Whitaker IS Rao J Izadi D Butler PE. Historical article: *Hirudo medicinalis*: ancient origins of, and trends in the use of medicinal leeches throughout history. Br J Oral Maxillofac Surg. (2004) 42:133–7. doi: 10.1016/S0266-4356(03)00242-0, PMID: 15013545

[16] Whitaker IS Izadi D Oliver DW Monteath G Butler PE. Hirudo Medicinalis and the plastic surgeon. Br J Plast Surg. (2004) 57:348–53. doi: 10.1016/j.bjps.2003.12.016, PMID: 15145739

[17] Whitaker IS Oboumarzouk O Rozen WM Naderi N Balasubramanian SP Azzopardi EA. The efficacy of medicinal leeches in plastic and reconstructive surgery: A systematic review of 277 reported clinical cases. Microsurgery. (2012) 32:240–50. doi: 10.1002/micr.20971, PMID: 22407551

[18] Kermanian CS Buote NJ Bergman PJ. Medicinal leech therapy in veterinary medicine: A retrospective study. J Am Anim Hosp Assoc. (2022) 58:303–8. doi: 10.5326/JAAHA-MS-7146, PMID: 36315858

[19] Bauters TGM Buyle FMA Verschraegen G Vermis K Vogelaers D Claeys G. Infection risk related to the use of medicinal leeches. Pharm World Sci. (2007) 29:122–5. doi: 10.1007/s11096-007-9105-3, PMID: 17353971

[20] Bauters T Buyle F Blot S Robays H Vogelaers D van Landuyt K. Prophylactic use of levofloxacin during medicinal leech therapy. Int J Clin Pharm. (2014) 36:995–9. doi: 10.1007/s11096-014-9986-x, PMID: 25097067

[21] Abdualkader M Merzouk A Ghawi AM Alaama AM. Some biological activities of Malaysian leech saliva extract. IIUM Engineering Journal. (2011) 12:1–9.

[22] Tasiemski A Vandenbulcke F Mitta G Lemoine J Lefebvre C Sautière P-E. Molecular characterization of two novel antibacterial peptides inducible upon bacterial challenge in an annelid, the leech *Theromyzon tessulatum*. J Biol Chem. (2004) 279:30973–82. doi: 10.1074/jbc.M312156200, PMID: 15102860

[23] Amachawadi RG Scott HN Nitikanchana S Vinasco J Tokach MD Dritz SS. Nasal carriage of mecA-positive methicillin-resistant Staphylococcus aureus in pigs exhibits dose–response to zinc supplementation. Foodborne Pathog. Dis. (2015) 12:159–163. doi: 10.1089/fpd.2014.1851 25551258

